# A Risk Prediction Model (CMC-AKIX) for Postoperative Acute Kidney Injury Using Machine Learning: Algorithm Development and Validation

**DOI:** 10.2196/62853

**Published:** 2025-04-09

**Authors:** Ji Won Min, Jae-Hong Min, Se-Hyun Chang, Byung Ha Chung, Eun Sil Koh, Young Soo Kim, Hyung Wook Kim, Tae Hyun Ban, Seok Joon Shin, In Young Choi, Hye Eun Yoon

**Affiliations:** 1 Department of Internal Medicine Bucheon St. Mary’s Hospital, College of Medicine The Catholic University of Korea Seoul Republic of Korea; 2 School of Information University of California Berkley, CA United States; 3 Department of Medical Informatics College of Medicine The Catholic University of Korea Seoul Republic of Korea; 4 Department of Internal Medicine Seoul St. Mary’s Hospital, College of Medicine The Catholic University of Korea Seoul Republic of Korea; 5 Department of Internal Medicine Yeouido St. Mary’s Hospital College of Medicine The Catholic University of Korea Seoul Republic of Korea; 6 Department of Internal Medicine Uijeongbu St. Mary’s Hospital, College of Medicine The Catholic University of Korea Seoul Republic of Korea; 7 Department of Internal Medicine St. Vincent’s Hospital, College of Medicine The Catholic University of Korea Seoul Republic of Korea; 8 Department of Internal Medicine Eunpyeong St. Mary’s Hospital, College of Medicine The Catholic University of Korea Seoul Republic of Korea; 9 Department of Internal Medicine Incheon St. Mary’s Hospital, College of Medicine The Catholic University of Korea Seoul Republic of Korea; 10 Department of Medical Informatics Graduate School of Healthcare Management & Policy, College of Medicine The Catholic University of Korea Seoul Republic of Korea

**Keywords:** acute kidney injury, general surgery, deep neural networks, machine learning, prediction model, postoperative care, surgery, anesthesia, mortality, morbidity, retrospective study, cohort analysis, hospital, South Korea, logistic regression, user-friendly, patient care, risk management, artificial intelligence, digital health

## Abstract

**Background:**

Postoperative acute kidney injury (AKI) is a significant risk associated with surgeries under general anesthesia, often leading to increased mortality and morbidity. Existing predictive models for postoperative AKI are usually limited to specific surgical areas or require external validation.

**Objective:**

We proposed to build a prediction model for postoperative AKI using several machine learning methods.

**Methods:**

We conducted a retrospective cohort analysis of noncardiac surgeries from 2009 to 2019 at seven university hospitals in South Korea. We evaluated six machine learning models: deep neural network, logistic regression, decision tree, random forest, light gradient boosting machine, and naïve Bayes for predicting postoperative AKI, defined as a significant increase in serum creatinine or the initiation of renal replacement therapy within 30 days after surgery. The performance of the models was analyzed using the area under the curve (AUC) of the receiver operating characteristic curve, accuracy, precision, sensitivity (recall), specificity, and *F*_1_-score.

**Results:**

Among the 239,267 surgeries analyzed, 7935 cases of postoperative AKI were identified. The models, using 38 preoperative predictors, showed that deep neural network (AUC=0.832), light gradient boosting machine (AUC=0.836), and logistic regression (AUC=0.825) demonstrated superior performance in predicting AKI risk. The deep neural network model was then developed into a user-friendly website for clinical use.

**Conclusions:**

Our study introduces a robust, high-performance AKI risk prediction system that is applicable in clinical settings using preoperative data. This model’s integration into a user-friendly website enhances its clinical utility, offering a significant step forward in personalized patient care and risk management.

## Introduction

Acute kidney injury (AKI) represents a critical challenge in postoperative care, significantly affecting patient outcomes and health care systems. It is a common complication that affects up to 5% to 7.5% of all hospitalized patients, with a markedly higher prevalence of 20% in intensive care units [1]. Among all AKI in hospitalized patients, 40% occur in postoperative patients [1]. This condition not only escalates morbidity but also substantially increases in-hospital mortality by approximately 3- to 9-fold [2]. The severity of this risk is further underscored in patients who developed postoperative AKI after intraabdominal surgery, as a large-scale study reported a 15-fold higher risk of mortality in patients with AKI compared to those without AKI [3]. Moreover, even patients whose renal function completely recovered after postoperative AKI still faced a higher risk of death compared to those without AKI [4,5], highlighting the profound and lasting consequences of this condition. These statistics underscore the need for accurate prediction and preemptive management of AKI in the postoperative setting.

There are many factors associated with postoperative AKI: age; sex; obesity; type of surgery; medications including renin-angiotensin-aldosterone system inhibitors (RASi) and nonsteroidal anti-inflammatory drugs (NSAIDs); and comorbidities such as chronic kidney disease (CKD), diabetes, hypertension, cardiovascular disease, liver disease, and chronic obstructive pulmonary disease [6-8]. These factors need to be integrated to assess the risk of postoperative AKI before surgery, and accurate risk prediction enables recognition of patients who need preoperative, intraoperative, and postoperative management to alleviate the risk. Several risk-scoring tools for postoperative AKI have been described [9-11]. However, their limitations are the homogeneity of the study population, the inclusion of a single center or a small number of centers, and the lack of external validation. To make the risk-scoring system generalizable, validation from a larger cohort using a multicenter database is needed [12]. Machine learning allows greater insight into possible interactions between variables and searches for as many informative and interesting feature relationships as possible, including those in subgroups, which can discover new variables involved in the event and is useful in a large dataset [13]. Therefore, the aim of this study was to build a risk prediction model for postoperative AKI using machine learning methods from a multicenter cohort.

## Methods

### Study Population

**Figure 1:**
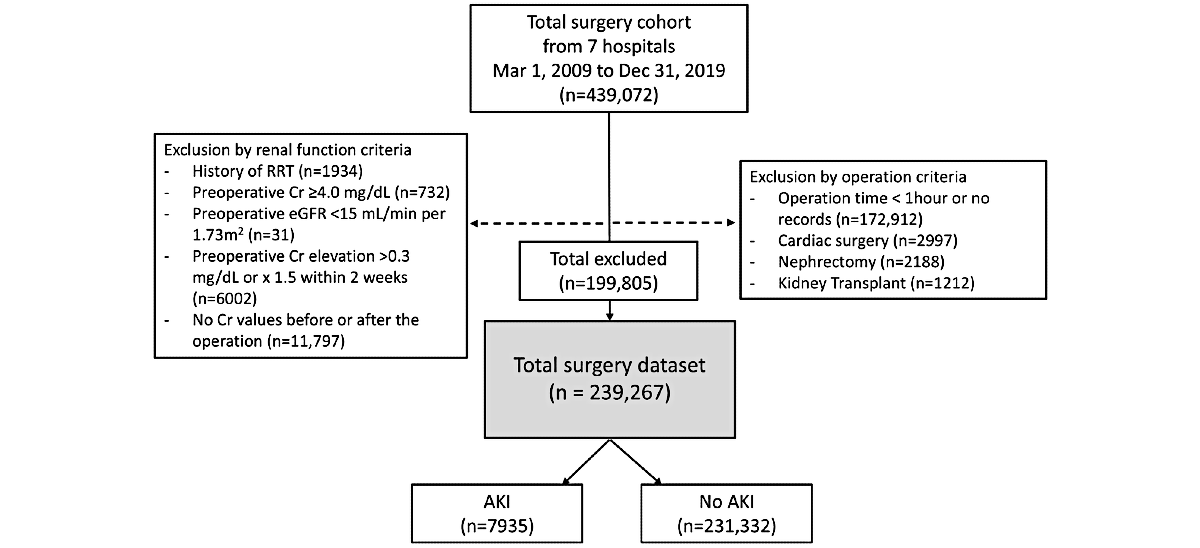
Flowchart of the study population. AKI: acute kidney injury; CMC: Catholic Medical Center; Cr: serum creatinine; eGFR: estimated glomerular filtration rate; op: operation; RRT: renal replacement therapy.

Patients who underwent general anesthesia surgery from March 1, 2009, to December 31, 2019, at seven academic hospitals of the Catholic University of Korea (Seoul St. Mary’s, Yeouido St. Mary’s, Uijeongbu St. Mary’s, Eunpyeong St. Mary’s, Bucheon St. Mary’s, St. Vincent, and Incheon St. Mary’s Hospitals) were included. The exclusion criteria were as follows: operation-related criteria were operation duration under 1 hour or duration not available, cardiac surgeries, operations of brain death donors, nephrectomies, and kidney transplant operations; and renal function-related exclusion criteria were patients with a history of renal replacement therapy, preoperative serum creatinine (sCr) ≥4.0 mg/dL or estimated glomerular filtration rate (eGFR) <15 mL/min per 1.73 m^2^, elevation of preoperative sCr more than 0.3 mg/dL or 1.5 times within 2 weeks before surgery, and patients without preoperative or postoperative sCr values (Figure 1).

### Ethical Considerations

The study was approved by the institutional review board of the Catholic University of Korea, College of Medicine (XC20WIDI0080) with waiver of consent due to the retrospective study methods. This study was not registered as it is a retrospective observational study. This report has been written according to the recently updated TRIPOD+AI (Transparent Reporting of a Multivariable Prediction Model for Individual Prognosis or Diagnosis+Artificial Intelligence) statement [14].

### Definition of Postoperative AKI

Postoperative AKI was defined as AKI that developed within 30 days after surgery, using the Kidney Disease: Improving Global Outcomes (KDIGO) criteria [15]. Stage-1 AKI was defined as sCr 1.5 to 1.9 times above baseline or an increase in sCr ≥0.3 mg/dL; stage-2 AKI was defined as sCr 2.0 to 2.9 times above baseline; and stage-3 AKI was defined as sCr more than 3 times above baseline, ≥4 mg/dL, or the initiation of renal replacement therapy (hemodialysis, peritoneal dialysis, or continuous renal replacement therapy). We did not use the urine output criteria of KDIGO, as previous studies suggested that the threshold of oliguria for postoperative AKI may be different from those of other AKIs [16,17] and due to the lack of data. This definition of postoperative AKI was used to create the supervised learning dataset of those with or without postoperative AKI.

### Data Collection and Cleansing

We collected data on demographic characteristics; underlying clinical diseases; preoperative laboratory data; preoperative medication; and surgical characteristics such as expected operation time, the day of operation (weekday or weekend), and the department of surgery. The underlying diseases of subjects were determined using the *International Statistical Classification of Diseases and Related Health Problems, 10th Revision* (*ICD-10*) codes of principal and secondary diagnosis. Comorbid diseases and *ICD-10* codes are shown in Multimedia Appendix 1. Preoperative medications included RASi (angiotensin converting enzyme inhibitor [ACEi] or angiotensin II type 1 receptor blocker [ARB]) or NSAIDs. Preoperative eGFR was calculated from the Chronic Kidney Disease Epidemiology Collaboration equation [18]. BMI was calculated as the patient’s weight in kilograms divided by height in meters squared (kg/m^2^).

Anonymized data was extracted from the Catholic Medical Center (CMC) Clinical Data Warehouse, which is separately generated and managed redundantly from the electronic medical record systems of eight affiliated hospitals of the College of Medicine, the Catholic University of Korea [5] and processed using R software (version 3.6.3; R Foundation for Statistics Computing). The 38 variables included in the final analysis are shown in Table 1. In cases where laboratory tests were conducted multiple times before surgery, we selected the most recent preoperative values, taken closest to the time of surgery, to ensure the data accurately reflected the patient’s latest clinical status. Data artifacts and extreme values were set to the 1st percentile and 99th percentile, and missing values were filled using multiple imputation by chained equations (MICE) [19]. MICE was used to provide more accurate estimates of the missing variables with the correlation of missing variables to other existing data points [20]. We excluded variables with more than 40% missing data, following common practice [21-23]. The rates of the missing data for the variables are shown in Multimedia Appendix 2. Nonbinary data were one-hot encoded, a method for rearranging categorical data into binary variables, and numerical data were normalized using min-max scaling. This would convert all numeric values between or equal to a value of 0 and 1. Min-max scaling is given by:



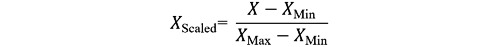



One-hot encoding, min-max scaling, and dataset splitting were accomplished using the *Scikit-Learn* library (version 0.24.2) [24]. These steps are required to improve the performance of machine learning models and training stability. Because there was a small percentage of AKI events (3.3%), there was an extreme class imbalance in the dataset. Such imbalances can cause a falsely elevated accuracy and adversely affect machine learning training [25]. To help overcome this issue, the AKI training dataset was augmented using an oversampling method by synthetic minority over-sampling technique (SMOTE), which has been shown to improve imbalanced class classifications (using *imblearn* library version 0.8.0) [26,27].

**Table 1:** Variables included in the final analysis.

Parameters	Variables
Patient parameters	Age Sex Systolic BP^a^ Diastolic BP BMI Chronic kidney disease Diabetes Hypertension Cerebrovascular disease Coronary artery disease COPD^b^ Liver cirrhosis Smoking Preoperative ACEi^c^ or ARB^d^ usage Preoperative NSAID^e^ usage
Surgical parameters	Department Weekday Operation duration
Laboratory parameters	White blood cell count Hemoglobin C-reactive protein Glucose Urea nitrogen Creatinine eGFR^f^ Total protein Albumin AST^g^ ALT^h^ Sodium Potassium Chloride Calcium Uric acid Creatine phosphokinase Lactic dehydrogenase Urine specific gravity Urine protein

^a^BP: blood pressure.

^b^COPD: chronic obstructive pulmonary disease.

^c^ACEi: angiotensin-converting enzyme inhibitor.

^d^ARB: angiotensin II type 1 receptor blocker.

^e^NSAID: nonsteroidal anti-inflammatory drug.

^f^eGFR: estimated glomerular filtration rate.

^g^AST: aspartate aminotransferase.

^h^ALT: alanine aminotransferase.

### Machine Learning

Various machine learning methods were used to create the model, which was trained and evaluated using Python (version 3.8.5; Python Software Foundation). Machine learning methods commonly used in health care were applied [28,29]. Models applied were logistic regression, decision tree, random forest, naïve Bayes (using *Scikit-Learn* library version 0.24.2) [24], light gradient boosting machine (GBM; using *lightgbm* version 3.2.1) [30], and deep neural network (DNN; using *Keras* library version 2.5.0) [31]. The strengths and weaknesses of each model have been summarized in Table 2.

**Table 2:** Characteristics of machine learning methods.

Method	How it works	Advantages	Disadvantages
DNN^a^ [32]	Multiple layers of interconnected nodes (neurons) of at least 3 hidden layers or more. Each neuron is a weighted sum of inputs and produces output by an activation function. Learns by backpropagation.	Can capture complex relationships between features, especially in larger datasets Can capture hierarchical features	Requires large amounts of data to avoid overfitting Computationally expensive
Logistic regression [33]	Linear classification algorithm that finds relationships between independent variables and a binary outcome using the probability from logistical functions.	Computationally less intensive Large datasets can be reasonably adapted	May not capture complex relationships between features
Decision tree [34]	A number of nodes that separate features depending on feature values and continue at each node, representing a tree.	Can capture complex relationships between features, especially in larger datasets	Prone to overfitting of data Can produce a biased decision tree depending on features
Random forest [35]	An ensemble (group) of decision trees that randomly select features and data for training, with decisions made by the ensemble using regression or other methods.	Can capture complex relationships between features, especially in larger datasets Less likely to overfit compared to a single decision tree	Can be computationally expensive
Light GBM^b^ [30]	An ensemble (group) of “weak models” (usually decision trees), which are sequentially added to one another to help to improve performance over a number of iterations.	Can capture complex relationships between features, especially in larger datasets Can capture more complex relationships compared to random forest	Can possibly overfit data
Naïve Bayes [36]	Makes use of conditional probability to represent the likelihood of classification given a certain set of features, assuming that each feature is independent of one another.	Computationally less intensive Large datasets can be reasonably adapted	May not capture complex relationships between features Relies on the assumption that features are independent of one another

^a^DNN: deep neural network.

^b^GBM: gradient boosting machine.

For the deep learning model, the structure that was chosen was a model that had an input layer of width 50 (to account for the 40 inputs and to include the one-hot encodings); 3 hidden layers with a width of 64, 32, and 32; and a single output node. The configurations of the different models are shown in Multimedia Appendix 3. The training of the models was done on a machine with Intel Xeon Gold 6240R (8 cores) at 2.40 GHz, with 64 GB of RAM, Windows 10 Enterprise Build 17763. To analyze the statistical performance of the models for postoperative AKI prediction, we assessed the area under the curve (AUC) of the receiver operating characteristic (ROC) curve, accuracy, precision, sensitivity (recall), specificity, and *F*_1_-score. To determine the optimal thresholds for ROC-AUC analysis and the calculation of sensitivity and specificity, we used the Youden index (Youden J statistic). This index is defined as:



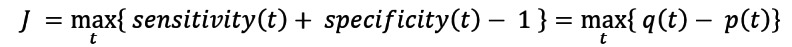



Alternatively, it can be expressed as the maximum value of *true positive rate – false positive rate*, which was the criterion applied in our models to identify the threshold value. This method ensures a balanced trade-off between sensitivity and specificity, as described in Schisterman et al [37].

### Statistical Analysis

Statistical analysis was performed using SAS software (version 9.4; SAS Institute). Continuous variables were presented as means and SDs for data with normal distribution and presented as medians and IQRs for data with nonparametric distribution. After the distribution of data between the two groups was determined, they were compared using an independent *t* test (2-tailed) or Wilcoxon rank sum test. Categorical data were presented as percentages, and a comparison between the two groups was performed using the chi-square test or Fisher exact test. To determine the risk factors for AKI, we used the logistic regression model. Multivariable analysis using logistic regression was performed on variables with a *P* value <.20 on univariable analysis [38]. The results are presented as odds ratio with 95% CI. *P* values <.05 were considered significant.

## Results

### Patient Baseline Characteristics

A total of 439,072 surgery cases from seven academic hospitals of the Catholic University of Korea were included in the study (Figure 1). After the exclusion of patients according to the exclusion criteria mentioned above, a total of 239,267 cases were included in the final analysis. Among these, 7935 (3.3%) AKI events occurred. Baseline demographics of patients with and without AKI are shown in Table 3. Significant differences were observed in all baseline characteristics between the two groups. Patients with AKI were older, with a higher percentage of those of the female sex, and had a lower BMI, a higher percentage of smokers, and higher baseline systolic and diastolic blood pressure. The AKI group also showed a higher percentage of all preexisting comorbidities (CKD, diabetes, hypertension, coronary artery disease, cerebrovascular disease, chronic obstructive pulmonary disease, or liver cirrhosis); more frequent usage of RASi (ACEi or ARB) and NSAIDs; and a higher percentage of patients undergoing general surgery, neurosurgery, and thoracic surgery. Laboratory results of the AKI group showed lower levels of hemoglobin, serum albumin, and eGFR, and higher baseline sCr and C-reactive protein levels. Variable selection was performed based on these clinical characteristics using logistic regression (clinical parameters are shown in Table 4 and laboratory parameters are shown in Table 5). During feature selection, the hematocrit variable was removed because it had >0.9 correlation with preoperative hemoglobin levels.

**Table 3:** Baseline characteristics.

Variables		No AKI^a^ (n=231,332)	AKI (n=7935)	*P* value	
Age (years), mean (SD)		54.56 (5.16)	61.36 (4.19)	<.001	
Male sex, n (%)		126,217 (54.6)	2997 (37.8)	<.001	
BMI (kg/m^2^), mean (SD)		24.29 (4.06)	24.07 (4.22)	<.001	
Smoker, n (%)		34,228 (14.8)	1424 (18.0)	<.001	
**Preexisting comorbidities**					
	Chronic kidney disease, n (%)	298 (0.1)	114 (1.4)	<.001	
	Diabetes, n (%)	7295 (3.2)	2663 (8.4)	<.001	
	Hypertension, n (%)	5802 (2.5)	504 (6.4)	<.001	
	Coronary artery disease, n (%)	3927 (1.7)	308 (3.9)	<.001	
	Cerebrovascular disease, n (%)	11,960 (5.2)	734 (9.3)	<.001	
	COPD^b^, n (%)	952 (0.4)	85 (1.1)	<.001	
	Liver cirrhosis, n (%)	1077 (0.5)	131 (1.9)	<.001	
	Systolic BP^c^ (mm Hg), mean (SD)	125.84 (15.74)	133.81 (20.7)	<.001	
	Diastolic BP (mm Hg), mean (SD)	69.71 (9.99)	67.01 (12.69)	<.001	
**Medication, n (%)**					
	ACEi^d^ or ARB^e^	7844 (3.4)	768 (9.7)	<.001	
	NSAIDs^f^	35,006 (15.1)	1650 (20.8)	<.001	
**Department, n (%)**					<.001
	General surgery	61,948 (26.8)	2409 (30.4)		
	Neurosurgery	31,169 (13.5)	1407 (17.7)		
	Orthopedics	60,442 (26.1)	1176 (14.8)		
	Obstetrics and Gynecology	22,607 (9.8)	299 (3.8)		
	Otorhinolaryngology	11,551 (5.0)	148 (1.9)		
	Thoracic surgery	10,774 (4.7)	427 (5.4)		
	Others	32,831 (14.2)	2069 (26.1)		
**Preoperative laboratory results, mean (SD)**					
	Hemoglobin (g/dL)	13.14 (1.83)	12.1 (2.15)	<.001	
	Urea nitrogen (mg/dL)	14.69 (5.47)	16.86 (8.75)	<.001	
	Creatinine (mg/dL)	0.81 (0.24)	0.91 (0.43)	<.001	
	eGFR^g^ (mL/min/1.73 m^2^)	95.41 (23.22)	82.75 (7.12)	<.001	
	Albumin (g/dL)	4.16 (0.51)	3.67 (0.69)	<.001	
	C-reactive protein (g/dL)	4.04 (18.35)	8.4 (26.4)	<.001	

^a^AKI: acute kidney injury.

^b^COPD: chronic obstructive pulmonary disease.

^c^BP: blood pressure.

^d^ACEi: angiotensin-converting enzyme inhibitor.

^e^ARB: angiotensin II type 1 receptor blocker.

^f^NSAID: nonsteroidal anti-inflammatory drug.

^g^eGFR: estimated glomerular filtration rate.

**Table 4:** Logistic regression analysis comparing clinical parameters between patients with and without acute kidney injury.

Clinical parameters	Univariable analysis			Multivariable analysis	
	Odds ratio (95% CI)	*P* value^a^	Odds ratio (95% CI)		*P* value
Age (years)	1.034 (1.032-1.036)	<.001	1.021 (1.019-1.023)		<.001
Sex: male (reference: female)	0.505 (0.483-0.529)	<.001	0.690 (0.652-0.73)		<.001
Systolic BP^b^ (mm Hg)	1.023 (1.022-1.024)	<.001	1.013 (1.011-1.014)		<.001
Diastolic BP (mm Hg)	0.974 (0.972-0.977)	<.001	0.983 (0.981-0.985)		<.001
BMI (kg/m^2^)	0.986 (0.981-0.992)	<.001	1.011 (1.006-1.017)		<.001
Chronic kidney disease: yes (reference: no)	11.303 (9.098-14.043)	<.001	2.248 (1.728-2.925)		<.001
Diabetes mellitus: yes (reference: no)	2.800 (2.577-3.042)	<.001	1.161 (1.05-1.284)		.01
Hypertension: yes (reference: no)	2.636 (2.4-2.896)	<.001	1.210 (1.08-1.357)		.01
Cerebrovascular disease: yes (reference: no)	1.870 (1.729-2.022)	<.001	1.217 (1.118-1.326)		<.001
Coronary artery disease: yes (reference: no)	2.338 (2.078-2.632)	<.001	1.049 (0.917-1.199)		.49
COPD^c^: yes (reference: no)	2.62 (2.097-3.275)	<.001	1.136 (0.896-1.441)		.29
Liver cirrhosis: yes (reference: no)	4.147 (3.493-4.925)	<.001	1.316 (1.086-1.595)		.01
Smoking: active (reference: never)	1.329 (1.253-1.411)	<.001	1.191 (1.112-1.275)		<.001
Operation duration	1.223 (1.21-1.237)	<.001	1.164 (1.150-1.178)		<.001
Preoperative ACEi^d^ or ARB^e^ usage: yes (reference: no)	3.053 (2.825-3.3)	<.001	1.326 (1.216-1.447)		<.001
Preoperative NSAIDs^f^ usage: yes (reference: no)	1.472 (1.393-1.556)	<.001	1 (0.941-1.062)		.99

^a^All variables with *P* value <.05 in the univariate analysis were included in the multivariate analysis.

^b^BP: blood pressure.

^c^COPD: chronic obstructive pulmonary disease.

^d^ACEi: angiotensin-converting enzyme inhibitor.

^e^ARB: angiotensin II type 1 receptor blocker.

^f^NSAID: nonsteroidal anti-inflammatory drug.

**Table 5:** Logistic regression analysis comparing laboratory parameters between patients with and without acute kidney injury.

Laboratory parameters		Univariable analysis			Multivariable analysis	
		Odds ratio (95% CI)	*P* value^a^	Odds ratio (95% CI)		*P* value
**Preoperative serum variables**						
	Albumin	0.269 (0.26-0.278)	<.001	0.524 (0.489-0.561)		<.001
	Total protein	0.452 (0.44-0.464)	<.001	1.042 (0.998-1.089)		.06
	White blood cell count	1.015 (1.013-1.017)	<.001	1.005 (1.003-1.008)		<.001
	ALT^b^	1.002 (1.002-1.003)	<.001	1 (0.999-1.001)		.62
	AST^c^	1.003 (1.002-1.003)	<.001	1 (1-1.001)		.52
	Urea nitrogen	1.05 (1.047-1.053)	<.001	1.001 (0.997-1.005)		.63
	Calcium	0.433 (0.419-0.448)	<.001	1.003 (0.959-1.049)		.88
	Chloride	0.975 (0.968-0.982)	<.001	1.005 (0.997-1.013)		.20
	Creatine phosphokinase	1(1-1)	<.001	1 (1-1)		.12
	Creatinine	3.034 (2.846-3.234)	<.001	3.218 (2.871-3.607)		<.001
	eGFR^d^	1.004 (1.004-1.005)	<.001	1.012 (1.011-1.013)		<.001
	C-reactive protein	1.007 (1.006-1.007)	<.001	0.998 (0.997-0.999)		.01
	Glucose	1.007 (1.006-1.007)	<.001	1.002 (1.002-1.002)		<.001
	Hemoglobin	0.747 (0.738-0.756)	<.001	1 (0.958-1.043)		.99
	Potassium	0.539 (0.51-0.57)	<.001	0.755 (0.715-0.798)		<.001
	Lactic dehydrogenase	1.001 (1.001-1.001)	<.001	1 (1-1)		<.001
	Uric acid	1.099 (1.084-1.114)	<.001	1.078 (1.062-1.095)		<.001
	Sodium	0.892 (0.886-0.898)	<.001	0.98 (0.971-0.99)		<.001
**Preoperative urine variables**						
	Dipstick protein	2.002 (1.948-2.057)	<.001	1.369 (1.325-1.416)		<.001
	Specific gravity	0.118 (0.023-0.617)	.01	0.042 (0.009-0.196)		<.001

^a^All variables with *P* value <.05 in the univariate analysis were included in the multivariate analysis.

^b^ALT: alanine aminotransferase.

^c^AST: aspartate aminotransferase.

^d^eGFR: estimated glomerular filtration rate.

### Model Performance

**Figure 2:**
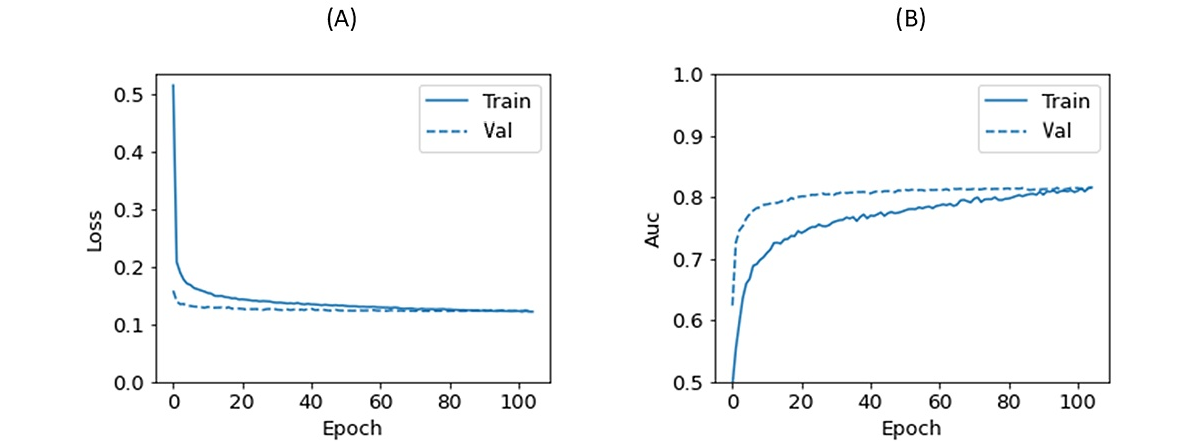
Loss function graph and AUC graph of the training and validation sets. (A) Loss functions of the validation and training sets converge at about epoch 86 and stabilize thereafter. (B) AUCs also overlap around epoch 86, and this suggests the model is starting to overfit on the training data. AUC: area under the curve; Val: validation.

The dataset was divided into the training set (80%) and the test set (20%). The training set (n=191,413) and test set (n=47,854) were balanced for outcomes and randomly assigned. To prevent outcome prediction bias, the testing subset was only evaluated after the model had been finalized. Predictive features were blinded during the outcome assessment phase. The loss function graph and AUC graphs of the training and validation sets for the DNN model are shown in Figure 2. Performance of the training and test sets of the DNN model is also presented in Multimedia Appendix 4. The performances of the different models are shown in Table 6.

**Figure 3:**
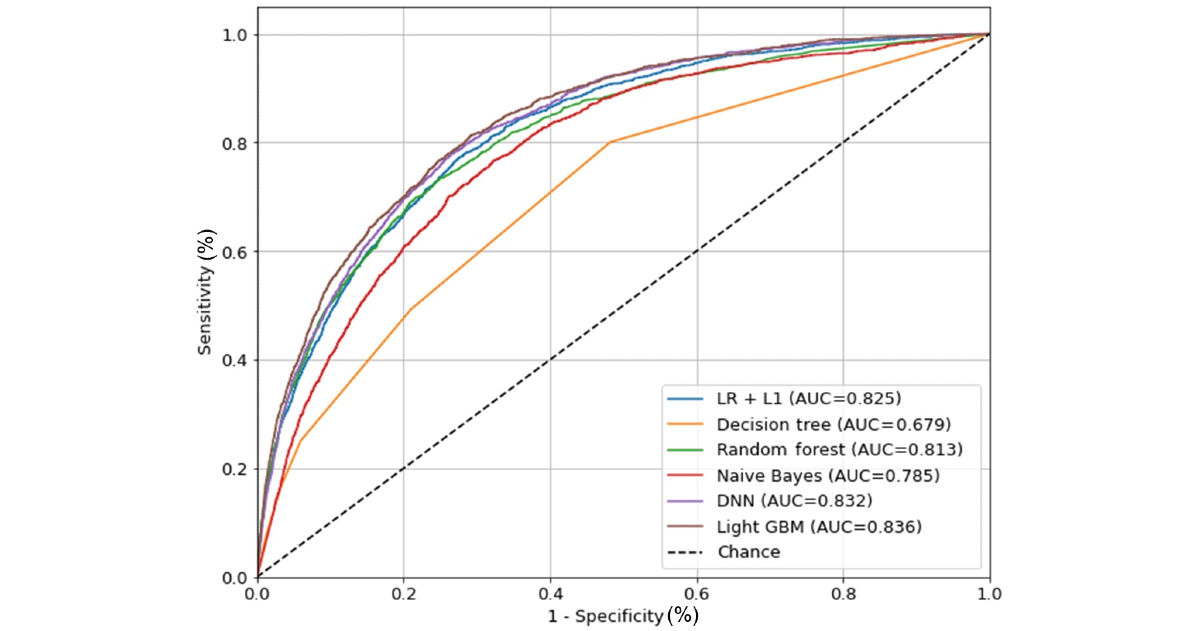
ROC-AUC of the AKI prediction models. AKI: acute kidney injury; AUC: area under the curve; DNN: deep neural network; Light GBM: light gradient boosting machine; LR + L1: logistic regression with Least Absolute Shrinkage and Selection Operator penalty; ROC: receiver operating characteristic.

We hypothesized that a simple system not using too many variables, for example, fewer than 20 variables, would be more practical to use in a clinical setting. Therefore, we evaluated model 2 and model 3 using multiple machine learning methods. Model 2 included 11 variables that were used in the classification system developed by Park et al [9], including age, sex, emergency operation, operation duration, diabetes, ACEi or ARB usage, blood levels of albumin, hemoglobin, sodium, eGFR, and urine dipstick protein. In this model, light GBM (AUC=0.81) and DNN (AUC=0.8) showed the highest performance. Model 3 included variables that were found significant on multivariable analysis, including age, sex, systolic blood pressure, diastolic blood pressure, operation duration, eGFR, blood levels of creatinine, albumin, sodium, potassium, chloride, glucose, lactic dehydrogenase, and urine dipstick protein. In this model as well, light GBM (AUC=0.825), and DNN (AUC=0.811) showed the highest performance. Model 1 included all 38 preoperative variables and surgical characteristics, on which light GBM (AUC=0.836), and DNN (AUC=0.832) demonstrated the best prediction performance once again. The ROC-AUC for model 1 of the different AKI prediction models is shown in Figure 3.

**Figure 4:**
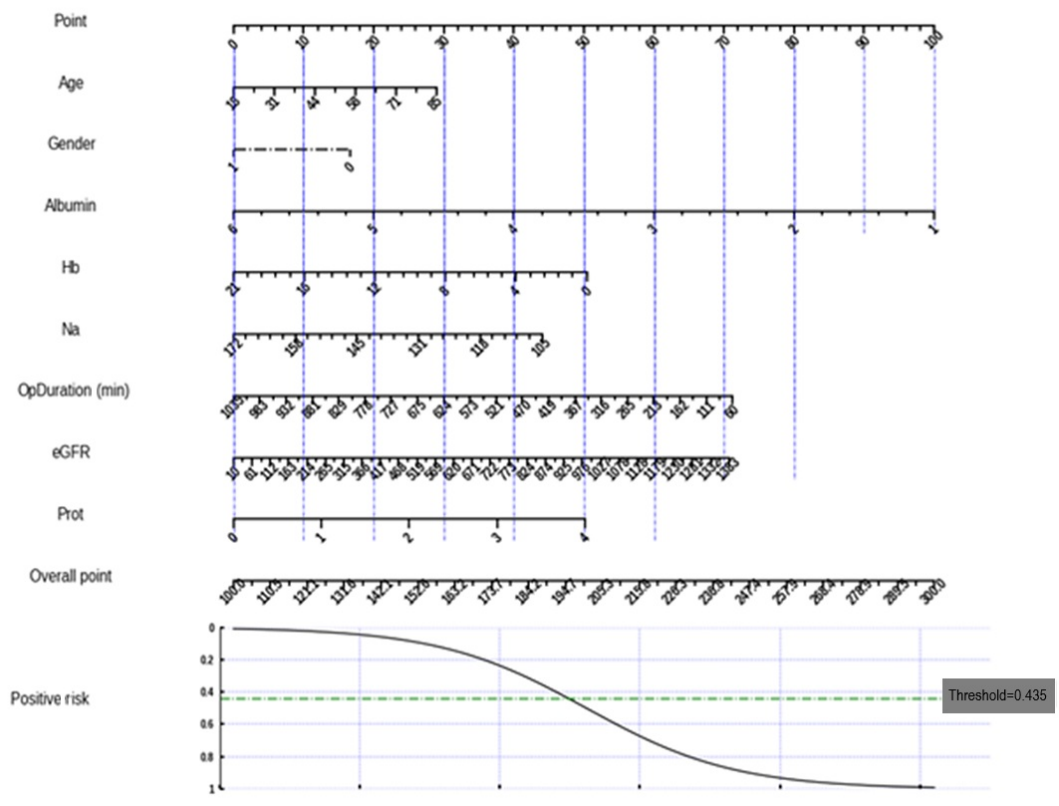
A nomogram based on a simplified logistic regression model. eGFR: estimated glomerular filtration rate; Hb: hemoglobin; Na: sodium; OpDuration: operation duration; Prot: urine protein.

To enhance clinical applicability, a nomogram was created based on a simplified logistic regression model, focusing on 8 key predictors: age, gender, albumin, hemoglobin, sodium, operation duration, eGFR, and urine protein. The nomogram was created by making modifications to a Python nomogram library called *simpleNomo* [39]. It provides a graphical tool for clinicians to estimate the risk of AKI in individual patients by integrating these factors. This approach allows quick risk stratification, clinical decision-making, and targeted interventions [40]. The nomogram is shown in Figure 4.

**Table 6:** Performance metrics of postoperative acute kidney injury prediction models.

Analysis and model			AUC^a^		Accuracy		NPV^b^		Precision or PPV^c^		Specificity		Recall or sensitivity	*F*_1_-score
**DNN^d^**														
	Model 1^e^	0.832		0.711		0.99		0.086		0.708		0.802		0.156
	Model 2^f^	0.8		0.712		0.988		0.082		0.711		0.75		0.147
	Model 3^g^	0.811		0.691		0.989		0.079		0.688		0.785		0.144
**Logistic regression**														
	Model 1	0.825		0.7		0.99		0.083		0.696		0.805		0.151
	Model 2	0.79		0.73		0.987		0.083		0.731		0.709		0.148
	Model 3	0.806		0.719		0.988		0.083		0.718		0.741		0.149
**Logistic regression with LASSO^h^ penalty**														
	Model 1	0.821		0.727		0.989		0.088		0.725		0.771		0.158
	Model 2	0.788		0.713		0.987		0.08		0.712		0.728		0.144
	Model 3	0.803		0.703		0.988		0.079		0.702		0.749		0.143
**Decision tree**														
	Model 1	0.679		0.606		0.983		0.056		0.603		0.691		0.104
	Model 2	0.711		0.635		0.984		0.062		0.633		0.708		0.114
	Model 3	0.626		0.844		0.976		0.085		0.86		0.379		0.139
**Random forest**														
	Model 1	0.813		0.751		0.988		0.092		0.752		0.732		0.163
	Model 2	0.806		0.708		0.988		0.081		0.706		0.751		0.146
	Model 3	0.812		0.756		0.987		0.091		0.758		0.708		0.161
**Light GBM^i^**														
	Model 1	0.836		0.711		0.991		0.087		0.708		0.813		0.157
	Model 2	0.81		0.73		0.988		0.086		0.73		0.74		0.154
	Model 3	0.825		0.728		0.988		0.087		0.727		0.756		0.156
**Naïve Bayes**														
	Model 1	0.785		0.68		0.988		0.075		0.677		0.767		0.137
	Model 2	0.773		0.662		0.988		0.072		0.658		0.77		0.131
	Model 3	0.792		0.742		0.986		0.086		0.744		0.701		0.153

^a^AUC: area under the curve.

^b^NPV: negative predictive value.

^c^PPV: positive predictive value.

^d^DNN: deep neural network.

^e^Model 1: age, sex, systolic blood pressure, diastolic blood pressure, BMI, chronic kidney disease, diabetes mellitus, hypertension, cerebrovascular disease, coronary artery disease, chronic obstructive pulmonary disease, liver cirrhosis, emergency operation, operation duration, angiotensin-converting enzyme inhibitor (ACEi) or angiotensin II type 1 receptor blocker (ARB) usage, nonsteroidal anti-inflammatory drug (NSAID) usage, estimated glomerular filtration rate (eGFR), blood levels of creatinine, total protein, albumin, aspartate aminotransferase (AST), alanine aminotransferase (ALT), urea nitrogen, sodium, potassium, chloride, calcium, creatine phosphokinase, lactic dehydrogenase, C-reactive protein, glucose, hemoglobin, and white blood cell count, urine specific gravity, and urine protein.

^f^Model 2: age, sex, emergency operation, operation duration, diabetes mellitus, ACEi or ARB usage, blood levels of albumin, hemoglobin, and sodium, eGFR, and urine protein.

^g^Model 3: age, sex, systolic blood pressure, diastolic blood pressure, operation duration, eGFR, blood levels of creatinine, albumin, sodium, potassium, chloride, glucose, and lactic dehydrogenase, and urine protein.

^h^LASSO: Least Absolute Shrinkage and Selection Operator.

^i^GBM: gradient boosting machine.

**Figure 5:**
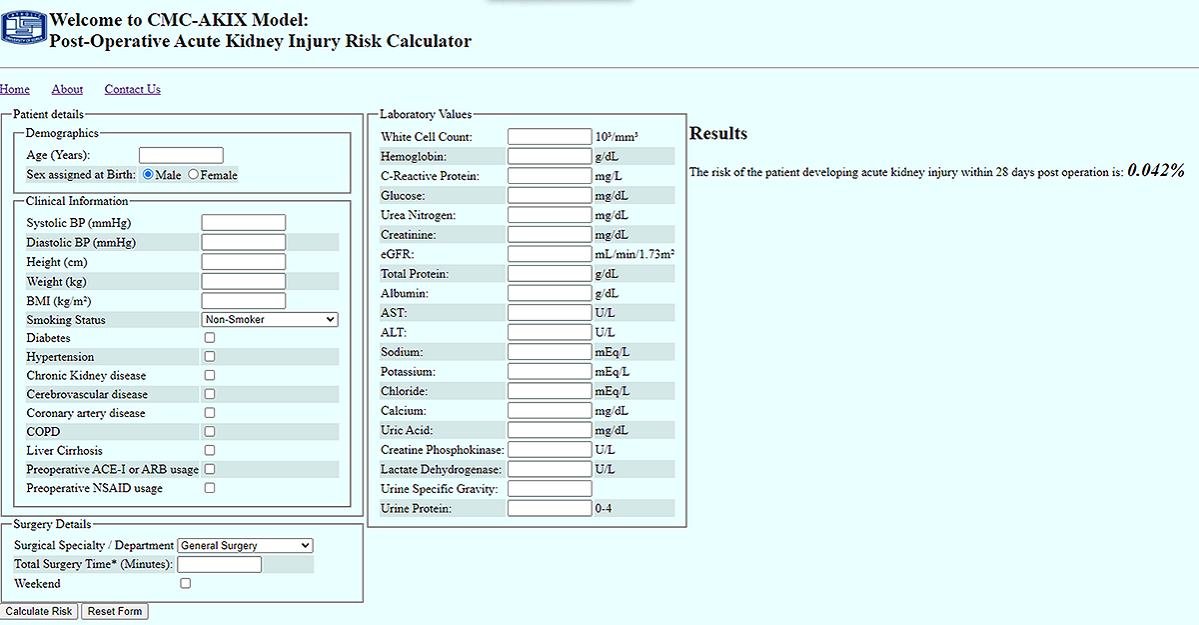
A sample page of the website application. ACE-I: angiotensin-converting enzyme inhibitor; ALT: alanine aminotransferase; ARB: angiotensin II type 1 receptor blocker; AST: aspartate aminotransferase; BP: blood pressure; CMC: Catholic Medical Center; COPD: chronic obstructive pulmonary disease; eGFR: estimated glomerular filtration rate; NSAID: nonsteroidal anti-inflammatory drugs.

Finally, our postoperative AKI prediction tool, the CMC-AKIX, was developed using all 38 variables. Therefore, the DNN model 1 was developed into a user-friendly website, which can be accessed on the web [41] (shown in Figure 5). This was created using Flask and hosted on a Google Cloud Virtual Machine.

## Discussion

Using a multicenter database of 239,267 noncardiac surgeries, we have developed a high-performance risk prediction system for postoperative AKI that can be easily applied. The model uses preoperative patient characteristics and laboratory data along with simple information about the surgery. DNN and light GBM showed a good performance in predicting postoperative AKI, with the best performance when all 38 variables were included.

AKI has a global presence and a high disease burden and mortality [42]. The incidence of AKI varies widely according to the geographic locations and is dependent on the setting: community acquired versus hospital acquired. It was reported that 1 in 5 adults and 1 in 3 children worldwide experience hospital-acquired AKI using the KDIGO definition [43]. Causes of hospital-acquired AKI include sepsis, critical illness, surgery, and use of nephrotoxic medications [44]. Postoperative AKI accounts for 30% to 40% of hospital-acquired AKI [1] and increases the risk of morbidity and in-hospital mortality [2]. Since treatment options are limited, the prevention of postoperative AKI is the cornerstone of improving patient outcomes after surgery [1]. Previous studies have found risk factors that increase the risk of postoperative AKI [1,6,7]. However, the definition of AKI using increased sCr levels as a marker of kidney damage has a limitation, because sCr levels begin to increase after the pathological changes of kidney injury are already in progress. Therefore, earlier and timely prevention and detection of postoperative AKI can be difficult [45]. This has led to continuous efforts to develop a risk stratification system for postoperative AKI. Recently, Park et al [9] have developed an index to classify postoperative AKI within 90 days after noncardiac surgery from 90,805 patients (SPARK index), which included 11 variables: age, sex, expected surgery duration, emergency operation, diabetes, use of RASi, baseline eGFR, dipstick albuminuria, hypoalbuminemia, anemia, and hyponatremia. The SPARK index showed a discrimination power of AUC of 0.80 for postoperative AKI in the discovery cohort and an AUC of 0.72 in the validation cohort.

Machine learning approaches are more flexible than statistical methods as they are free from statistical assumptions such as noncollinearity or normal distribution of residuals. It allows all possible interactions between variables according to multidimensional nonlinear patterns and aggressively searches for as many informative and interesting features as possible [13]. Lei et al [11] used machine learning techniques to stratify the risk of postoperative AKI within 7 days after noncardiac surgery from a single center cohort of 42,615 patients. In that study, GBM showed the highest performance with an AUC of 0.817 (95% CI 0.802-0.832) and included 339 preoperative and intraoperative variables. Bihorac et al [10] developed a machine learning–based risk prediction tool (MySurgeryRisk) for 8 major postoperative complications within 24 months after any kind of surgery from a single center cohort of 51,457 patients. Using this platform, the authors validated the model’s performance for predicting postoperative AKI, with an AUC of 0.82 (95% CI 0.82-0.83), including 135 variables from a cohort of 22,300 surgeries [46].

The strength of our study is that we used a multicenter dataset of a larger scale than previous ones. Data were extracted from the CMC Clinical Data Warehouse, which included data from seven academic hospitals located in five cities in South Korea. The prediction model was developed using 38 clinical and laboratory parameters in combination that exhibited the best prediction performance. These variables are used in clinical practice and can be extracted from electronic medical records. In addition, by including the department of surgery as a variable, the CMC-AKIX can be applied to various kinds of noncardiac surgery. We are looking to simplify the model and improve usability by allowing incomplete data or missing values to be filled in with best estimate values using imputation methods such as MICE.

Our study holds a distinct advantage in that it compared several of the most widely used machine learning methods in clinical data modeling. By doing so, we systematically observed and elucidated the strengths and limitations of each model, using a large, well-curated dataset. We observed that certain methods were more affected by the imbalanced dataset, including the decision tree classifier, random forest, and naïve Bayes. We aim to offer insights into the selection of different algorithms for applications in clinical studies.

This study has several limitations. First, the results of this study have not been externally validated in independent cohorts from different countries, races, and ethnicities. As such, further external validation is needed to assess the generalizability of the CMC-AKIX model across diverse populations. Second, our definition of postoperative AKI as AKI developing within 30 days after surgery may be controversial as most studies observing postoperative AKI apply the time period of 7 days in conjunction with the KDIGO criteria [15]. A 30-day period was selected for this study because postoperative complications or morbidity in most studies is defined as events occurring within 30 days after surgery [47,48]. Patients with AKI that persist for more than 7 days, beyond the 30-day period of the study, have been organized in a second cohort study observing postoperative risk of acute kidney disease and CKD [49]. Third, the urine output definition of the KDIGO criteria was not used because of a lack of urine output data. This could have led to incomplete identification of postoperative AKI. Fourth, the intraoperative and postoperative factors were not included in the risk prediction system, which also affects postoperative renal outcomes. As the purpose of our model is mainly to identify patients at high risk for postoperative AKI while they are still in the preoperative setting, intraoperative and postoperative variables should not be included.

In the future, we look to collaborating with other institutions with different demographic data to validate the model and see if it could perform well with different demographic populations. Also, the model will be fine-tuned in the process of including diverse datasets, and the performance of the model will be improved by creating an appropriate ensemble of machine learning models to gain the benefits of the different machine learning structures and advantages [50]. At last, practical use of the model may be significantly increased by incorporating it into an electronic alert system to automatically identify patients at high risk for postoperative AKI, providing timely risk alerts, and thereby allowing for proactive management such as cessation of causative medications or prescription of fluids—ultimately improving patient care [51]. Such systems could also allow for continuous updates and refinements of the model as new data become available, ensuring its relevance and adaptability. By supporting evidence-based decision-making and improving perioperative risk management, this approach has the potential to significantly enhance patient outcomes and optimize resource allocation in diverse health care settings. In conclusion, we propose a machine learning–based risk prediction tool, the CMC-AKIX, using individual patients’ preoperative characteristics and surgical information. This model was adapted to a user-friendly web-based program, and one can use it even if all variables are not included. This tool may guide preoperative counseling, decision-making, and perioperative care.

## Data Availability

Access to the datasets generated or analyzed in this study is available from the corresponding author upon reasonable request.

## Appendix

Multimedia Appendix 1 ICD-10 (International Statistical Classification of Diseases and Related Health Problems, 10th Revision) codes for comorbid conditions.

Multimedia Appendix 2 Rates of missing data for the variables before imputation.

Multimedia Appendix 3 Code and configurations of the deep neural network.

Multimedia Appendix 4 Performance of the training and test sets of the deep neural network model.

## Abbreviations

AKI: acute kidney injury
ACEi: angiotensin-converting enzyme inhibitor
ARB: angiotensin II type 1 receptor blocker
AUC: area under the curve
CKD: chronic kidney disease
DNN: deep neural network
GBM: gradient boosting machine
*ICD-10*: *International Statistical Classification of Diseases and Related Health Problems, 10th Revision*
eGFR: estimated glomerular filtration rate
KDIGO: Kidney Disease: Improving Global Outcomes
MICE: multiple imputation by chained equations
NSAID: nonsteroidal anti-inflammatory drug
RASi: renin-angiotensin-aldosterone system inhibitors
ROC: receiver operating characteristic
RRT: renal replacement therapy
sCr: serum creatinine
SMOTE: synthetic minority over-sampling technique
TRIPOD+AI: Transparent Reporting of a Multivariable Prediction Model for Individual Prognosis or Diagnosis+Artificial Intelligence
