# Supplementary material for: A Risk Prediction Model (CMC-AKIX) for Postoperative Acute Kidney Injury Using Machine Learning: Algorithm Development and Validation
Source: J Med Internet Res. 2025 Apr 9;27:e62853. doi: 10.2196/62853 (PMC12018867; doi:10.2196/62853)
Supplement: Multimedia Appendix 1 [file jmir_v27i1e62853_app1.docx]

**Multimedia Appendix 1. ICD-10 Codes for Comorbid Conditions.**

| **Comorbid condition** | **ICD-10 code** |
| --- | --- |
| Chronic kidney disease | N18, N19 |
| Diabetes mellitus | E10 ~ E14 |
| Hypertension | I10 ~ I15 |
| Cerebrovascular disease | I60 ~ I69 |
| Coronary artery disease | I20 ~ I25, I50, I51 |
| Chronic obstructive pulmonary disease | J44 |
| Liver cirrhosis | K74 |

ICD-10, International Classification of Disease, 10^th^ Revision.
