# Supplementary material for: A Risk Prediction Model (CMC-AKIX) for Postoperative Acute Kidney Injury Using Machine Learning: Algorithm Development and Validation
Source: J Med Internet Res. 2025 Apr 9;27:e62853. doi: 10.2196/62853 (PMC12018867; doi:10.2196/62853)
Supplement: Multimedia Appendix 2 [file jmir_v27i1e62853_app2.docx]

**Multimedia Appendix 2. Rates of Missing Data for the Variables before Imputation**


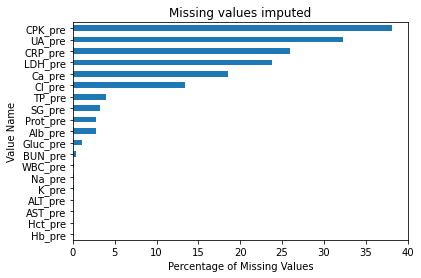


| Variable (full names) | Percentage of Missing Values (%) |
| --- | --- |
| CPK_pre (Creatine Phosphokinase)  UA_pre (Uric Acid)  CRP_pre (C-Reactive Protein)  LDH_pre (Lactate Dehydrogenase)  Ca_pre (Calcium)  Cl_pre (Chloride)  TP_pre (Total Protein)  SG_pre (Specific Gravity)  Prot_pre (Protein)  Alb_pre (Albumin)  Gluc_pre (Glucose)  BUN_pre (Blood Urea Nitrogen)  WBC_pre (White Blood Cell Count)  Na_pre (Sodium)  K_pre (Potassium)  ALT_pre (Alanine Aminotransferase)  AST_pre (Aspartate Aminotransferase)  Hct_pre (Hematocrit)  Hb_pre (Hemoglobin) | 38.119757  32.234700  25.922923  23.848253  18.551242  13.354119  3.901917  3.255777  2.789352  2.727915  1.070770  0.319390  0.158400  0.095709  0.083589  0.047228  0.025077  0.022151  0.020479 |
