# Supplementary material for: A Risk Prediction Model (CMC-AKIX) for Postoperative Acute Kidney Injury Using Machine Learning: Algorithm Development and Validation
Source: J Med Internet Res. 2025 Apr 9;27:e62853. doi: 10.2196/62853 (PMC12018867; doi:10.2196/62853)
Supplement: Multimedia Appendix 3 [file jmir_v27i1e62853_app3.docx]

**Multimedia Appendix 3. Code and Configurations of the Deep Neural Network.**

| - 1. **Machine learning model** |  |
| --- | --- |
| Logistic regression | C: 10  Class_Weight:“balanced”  Max_iter: 100  Penalty: “l1”  Solver” “liblinear” |
| Light GBM classifier | colsample_bytree: 0.45346047315892635  min_child_samples: 474  min_child_weight: 10.0  num_leaves: 45  reg_alpha: 0.1  reg_lambda: 100  subsample: 0.4108882768942656 |
| DNN | Structure:   - Input layer: - Hidden Layers: 3   - Density: 64, 32, 32   - Dropout: 0.5   - Activation Function: ReLU - Output Layer:   - Activation Function: Sigmoid   Optimizer: “Adam”  Loss Function: Binary Cross Entropy  Epoch: Set at 500, ended at 95 with early stopping  Early Stopping Function   - Monitor: “val_auc” - Patience: 10 - Mode: “max” - Restore_best_weights: True   Validation dataset: 20% of Training dataset |
| Decision tree | Criterion: “gini”  Max_depth: 3  Max_features: “auto”  Max_leaf_nodes: 10  Min_samples_leaf: 2  Min_samples_split: 7 |
| Random forest | Max_depth: “None”  Max_features: “auto”  Min_samples_leaf: 1  Min_samples_split: 5  N_ |
| Naïve Bayes | Priors: 0.999, 0.001 |

DNN: deep neural network; GBM: gradient boosting machine.
