# Supplementary material for: A Risk Prediction Model (CMC-AKIX) for Postoperative Acute Kidney Injury Using Machine Learning: Algorithm Development and Validation
Source: J Med Internet Res. 2025 Apr 9;27:e62853. doi: 10.2196/62853 (PMC12018867; doi:10.2196/62853)
Supplement: Multimedia Appendix 4 [file jmir_v27i1e62853_app4.docx]

**Multimedia Appendix 4. Performance of the Training and Test Sets** **of the DNN Model**

| **Parameters** | **Training Set** | **Validation Set** | **Test Set** |
| --- | --- | --- | --- |
| AUC | 0.843 | 0.815 | 0.832 |
| Accuracy | 0.767 | 0.737 | 0.711 |
| Precision | 0.101 | 0.090 | 0.086 |
| Specificity | 0.767 | 0.736 | 0.708 |
| Recall/Sensitivity | 0.767 | 0.756 | 0.802 |
| F1 score | 0.179 | 0.161 | 0.156 |

AUC, Area under curve
